# Collection of cell-free DNA for genomic analysis of solid tumors in a clinical laboratory setting

**DOI:** 10.1371/journal.pone.0176241

**Published:** 2017-04-27

**Authors:** Christopher K. Raymond, Jennifer Hernandez, Reynold Karr, Kay Hill, Mark Li

**Affiliations:** 1 Resolution Bioscience, Bellevue, Washington, United States of America; 2 PlasmaLab International, Everett, Washington, United States of America; 3 Dept. of Rheumatology, University of Washington, Seattle, Washington, United States of America; European Institute of Oncology, ITALY

## Abstract

The breadth of diagnostic procedures that utilize cell free DNA (cfDNA) from human plasma has increased dramatically in recent years. Here, we confirm that tumor-derived cfDNA fragments are similar in size distribution to cfDNA derived from normal tissues. Therefore, collection procedures optimized with healthy donor specimens are likely to be applicable to the diagnosis and monitoring of many different cancer types. We verify that the distribution and DNA sequences of fragmentation sites in cfDNA from both normal-germline and tumor-derived cfDNA are non-random. A broad survey of cfDNA from healthy donors suggests that average individuals possess ~6 ng of cfDNA per mL of plasma. Importantly, the cfDNA present in plasma samples that were initially collected as whole blood in K2-EDTA tubes and subsequently processed by centrifugation is stable for several days at ambient temperatures. This observation has the potential to significantly reduce the cost and logistical complexity of shipping clinical samples from the site of collection to centers proficient in diagnostic analysis. Finally, plasma samples collected with high-volume plasma collection devices possess abundant quantities of cfDNA. Since the quantity of analyzed cfDNA is directly proportional to sensitivity of diagnostic assays, this method of plasma collection, where available, could enable highly sensitive post-treatment disease monitoring and early detection of cancer in at-risk individuals.

## Introduction

The analysis of cfDNA found in the plasma fraction of whole blood is improving the quality of patient health care by providing non-invasive methods to monitor health and disease (reviewed in [1,2]). Following the discovery of fetal cfDNA in maternal blood [3], non-invasive prenatal testing has become the first-line diagnostic screening method for the detection of fetal trisomy. Similarly, it has been known for decades that many cancer patients possess circulating tumor DNA [1,2,4,5]. The development of next-generation sequencing (NGS) and its application to cfDNA has shown that many solid tumor types can be genotyped by sequencing of cell-free DNA [6]. As the development and application of targeted therapies used to treat cancers that have specific genetic alterations becomes more widespread, there is an increasing demand for robust, cost-effective, non-invasive tumor genotyping that can match patients with treatments that are likely to be effective [7]. Indeed, the first non-invasive, blood-based test for treatment of EGFR mutant non-small cell lung cancer (NSCLC) was recently approved by the FDA [8]. While this test is a PCR-based “liquid biopsy,” it points to a future where NGS is applied to blood-based detection, diagnosis and monitoring of solid tumors.

There are conflicting reports in the literature about the origin and composition of circulating tumor DNA. On the one hand, there are clear demonstrations that tumor DNA is present as nucleosome sized, ~150–180 bp fragments [9, 10]. Alternatively, several studies suggest that circulating tumor DNA is present as higher molecular weight DNA fragments shed from necrotic cells that have expired and burst [11,12]. Here we provide evidence that tumor-specific mutations are reliably detected in nucleosomal cfDNA fragments. We go on to suggest scalable, practical and cost-effective ways to collect and ship cfDNA from sites of collection to sites where genomic analysis is performed. Lastly, we demonstrate that large scale collection of cfDNA from single individuals can be achieved with existing technology.

## Materials and methods

### Ethics statement

The ethics committees of Resolution Bioscience and of Plasma Lab International reviewed and approved of the research presented here. Samples from cancer patients were provided by commercial biorepositories who obtained written consent from patients and who themselves provided written consent for use of these samples in this study. Written consent was obtained from healthy donors prior to sample collection, processing and characterization.

### Blood collection and processing

For studies done with K2 EDTA tubes, both patient and healthy volunteer samples were collected in 10 mL K2 EDTA BD Vacutainer tubes (Beckton Dickinson, Franklin Lakes, NJ). Clarified plasma was prepared from 10 mL tubes by centrifugation at 2000 x g for 10 min; transfer of the initial plasma fraction to a conical 15 mL centrifuge tube and a second 2000 x g spin for 10 min; and decanting of the clarified plasma, carefully avoiding the residual pellet material. Larger scale, 100 mL to 800 mL plasma collection was performed with Autopheresis-C System instruments from Fenwal, Inc. (Lake Zurich, IL) with collection into plasma collection bottles that contain Na_3_-citrate as an anticoagulant.

Cell-free DNA was purified from plasma using the Qiagen Circulating Nucleic acids kit (Qiagen, Hilden, Germany). The yield of double-strand DNA was quantified using a Qubit fluorometer (Thermo Fisher, Waltham, MA) and the corresponding hsDNA quantitation kit. Size analysis was performed using gel electrophoresis on 2% agarose gels with PCR markers as size standards (New England Biolabs, Ipswich, MA). The spike-in DNA used in this study was structural multiplex cfDNA reference standard, product HD786, from Horizon (Cambridge, UK). It was added to healthy donor plasma at a ratio of spike-in to total cfDNA of 1:10.

### Genomic analysis

Cloning, NGS and post-sequence analysis of cfDNA were performed using the proprietary Resolution Bioscience sample analysis pipeline. The central elements of sample analysis are illustrated in supplemental S1 Fig. Broadly speaking, this is a targeted hybrid capture technology [13,14] in which genomic libraries are constructed from cfDNA. The adaptors used to make these libraries carry unique molecular identifiers (UMIs) [15] that are used to identify non-redundant and redundant genomic clones. The adaptors also possess sequences that enable sample multiplexing. Hybrid capture is followed by primer extension of the probe sequence. This marks each genomic clone in the targeted sequencing library with the hybridizing probe that interacted with that clone. Samples were sequenced on a NextSeq NGS system from Illumina (San Diego, CA) using paired end sequencing with asymmetric reads lengths. Bioinformatic analysis was performed as described in a previous publication [14].

## Results

### Tumor DNA is found in nucleosomal fragments

**Fig 1:**
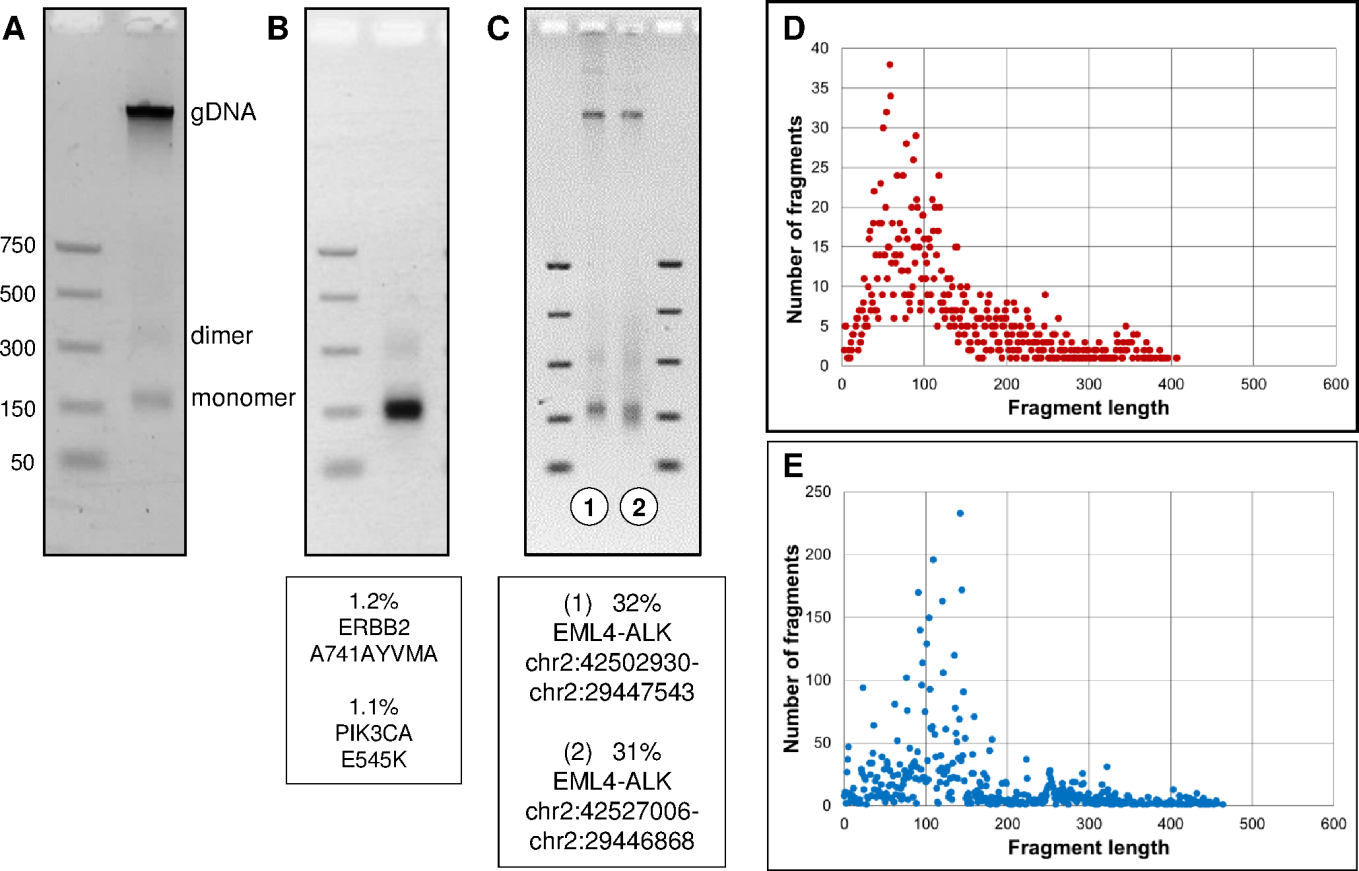
Circulating tumor DNA is detected in the nucleosomal fraction of cfDNA. (A) The DNA purified from plasma often consists of short nucleosome-sized monomer, dimer, trimer, etc. DNA fragments and high molecular weight gDNA. (B) The cfDNA extracted from the plasma of a patient with NSCLC appeared to be exclusively nucleosomal fragments, and tumor-specific mutations shown below the gel were detected in the resulting genomic analysis. (C) The cfDNA isolated from two NSCLC patients that had independent EML4-ALK fusions. Sequencing revealed that the tumor fraction (minor allele frequency of the EML4-ALK fusion) was 32% in the patient sample on the left and 31% in the patient sample shown on the right. (D) Size distribution of tumor derived DNA fragments (top) and germline DNA fragments (bottom). The EML4-ALK fusion tumor data was calculated by combining the unique read information from four independent fusion genes found in four separate NSCLC patients, including the two shown in Fig 1C.

Clinical laboratories that have the capacity to perform genomic analysis on cfDNA collected from cancer patients often receive frozen plasma for analysis. Generally, this plasma comes from whole blood that was collected in K2 EDTA tubes and centrifuged at the site of collection to separate the plasma from blood cells. The DNA isolated from many of these samples is composed of both nucleosome-sized monomers (~165 bp), dimers (330 bp), etc. and high molecular weight (≥5000 bp) genomic DNA (Fig 1A). In the majority of cases, the high molecular weight gDNA is partially or completely removed by an additional centrifugation step, suggesting the gDNA is contributed by nucleated cells that were not completely removed by centrifugation. Here, we demonstrate that tumor-derived DNA fragments reside in the nucleosomal fraction of cfDNA.

Next-generation DNA sequence analysis (NGS) was used to investigate the molecular characteristics of cfDNA. A brief outline of the sequencing technology that was used is shown in S1 Fig and is described in more detail in ref. [14]. In some cases, patient cfDNA samples appear to be exclusively nucleosomal fragments and minor somatic alleles indicative of tumor DNA are readily detected (Fig 1B). We extended this analysis a step further by looking at the size distribution of tumor-associated EML4-ALK fusion fragments relative to germline cfDNA (Fig 1C). In this analysis, the genomic coordinates of the DNA capture probes are fixed relative to a distribution of cfDNA fragmentation sites. Hence the aggregate analysis derived from many individual clones reveals a distribution of fragment sizes. The distribution of tumor-derived EML4-ALK fusion fragments and germline DNA fragments was highly similar, demonstrating that genomic DNA released from cancerous cells circulates in plasma as nucleosomal-sized fragments. The key point is that collection methods that emphasize the enrichment and analysis of nucleosome-sized DNA fragments are likely to generate test results that reflect the actual tumor DNA content of patient samples.

### cfDNA fragmentation is non-random

**Fig 2:**
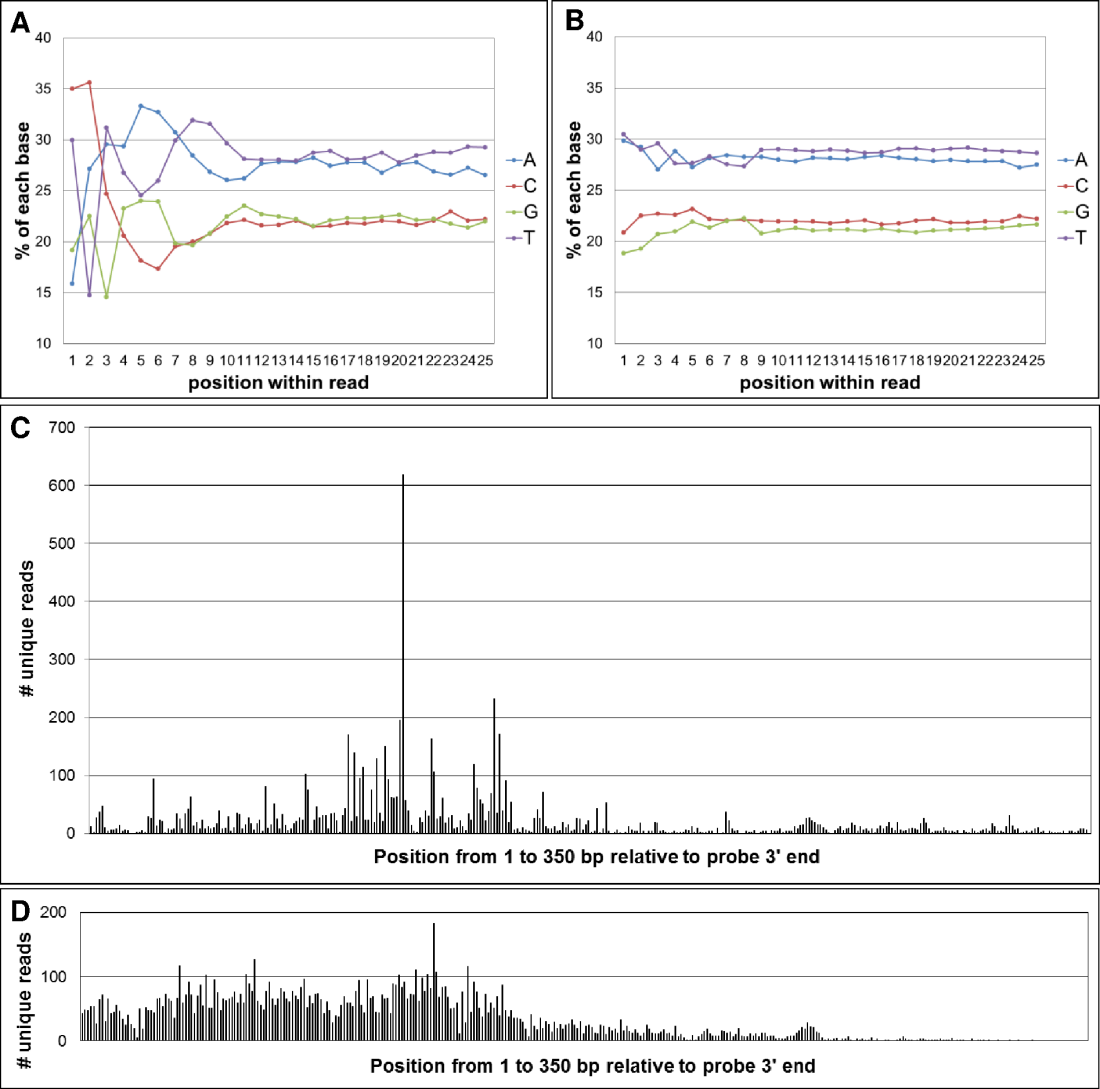
Comparison of cfDNA fragmentation with sonicated DNA fragmentation. (A) The base composition of the first 25 bases from 100,000 independent cfDNA clones. (B) The base composition of the first 25 bases from 100,000 independent sonicated-DNA clones. (C) The distribution of 7,781 unique cfDNA fragmentation sites across 350 base pairs. The 3’ end of the capture probe corresponds to base position 0. (D) The distribution of 11,384 unique sonicated fragmentation sites across the same 350 base pair segment shown in Fig 2C.

Sequence analysis of cfDNA clone ends revealed a biased, non-random distribution of bases in the first few nucleotides of cfDNA clones (Fig 2A). The first and second bases were dominated by cytosine. Additionally, there were pronounced deficits of adenine in the first base position, thymidine in the second base position, and guanosine in the third base position. In contrast, the clone end profile of sonicated DNA has very little bias (Fig 2B). Importantly, clone end analysis of the 2,281-independent tumor-associated EML4-ALK fusion fragments from four independent patients showed a nucleotide position bias profile similar to germline cfDNA (S2 Fig).

To explore the fragmentation pattern of cfDNA in more granular detail, we compared the first base starting position in healthy donor cfDNA relative to sonicated, size-selected genomic DNA (Fig 2C). Once again, the distribution frequency of clone ends at various genomic coordinates is shown relative to their interacting DNA capture probe that has a fixed genomic location. In this analysis, only unique, non-redundant, “de-duplicated” reads were considered. The observed clone-end-distribution-bias of cfDNA fragments shown in Fig 2C is both highly reproducible from sample to sample, and similar, highly biased cfDNA clone end distributions were found at other probe locations we analyzed (not shown). The core lesson for our laboratory was that the collection of sequence information from cfDNA in a clinical setting requires analysis methods that can anticipate and accommodate the non-random fragmentation features unique to cfDNA.

### Processing and shipping of cfDNA-containing plasma samples

**Fig 3:**
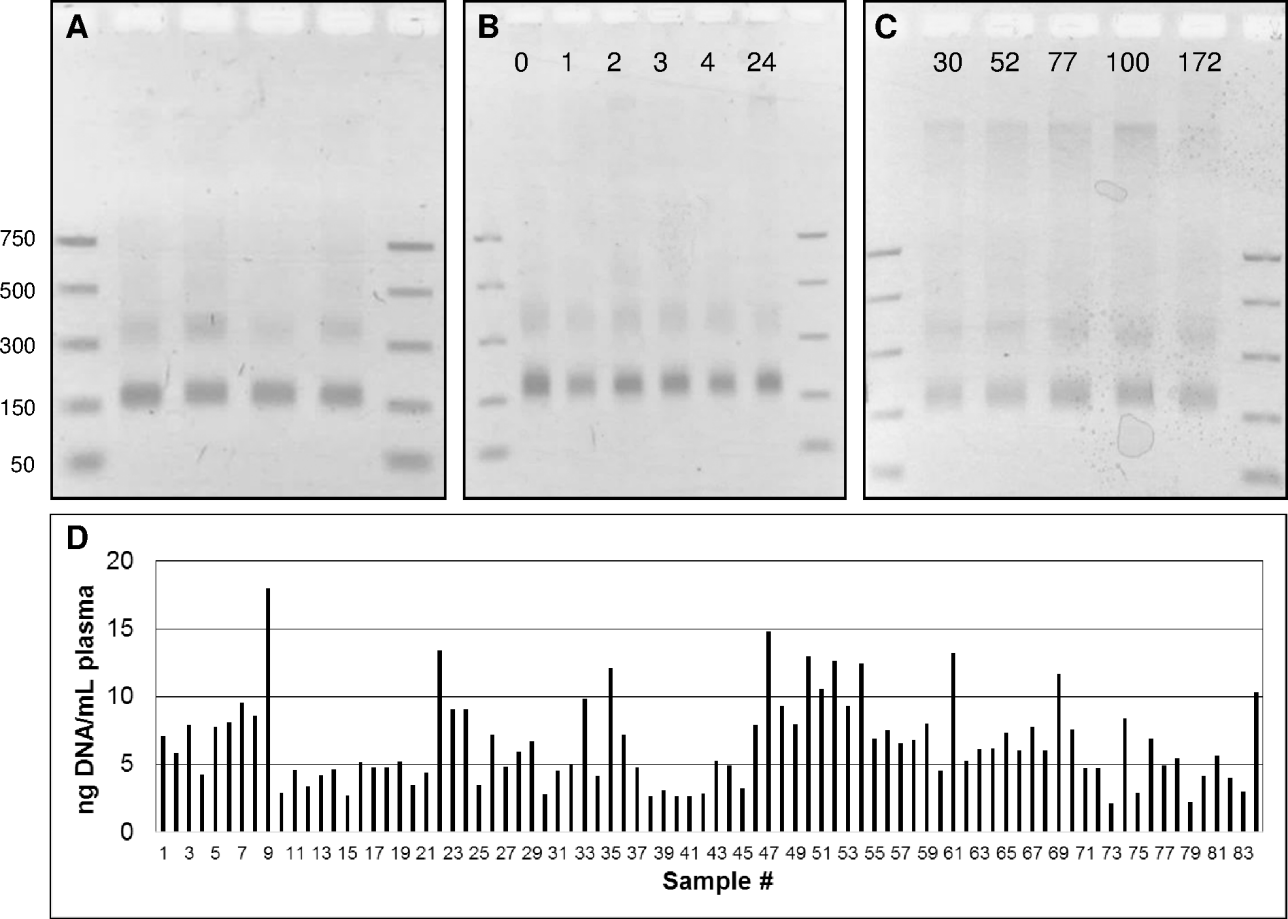
Analysis of cfDNA collected from healthy donors. (A) The cfDNA extracted from four representative healthy donors. (B) The cfDNA extracted from a single donor were processed from whole blood to plasma at the indicated times (hours). (C) The cfDNA extracted from plasma after storage then shipping of the plasma at the indicated times (hours). (D) The cfDNA yields from 84 healthy donors.

Having established that genomic analysis of nucleosomal DNA fragments was clinically informative, we wanted to determine the reference range of cfDNA yields in a healthy volunteer population. In addition, we wanted to explore pragmatic, economical and scalable methods to collect and transport clinical plasma samples whose intended use was genomic analysis. We focused on anticoagulant K2 EDTA vacutainer tubes because they are ubiquitous in phlebotomy laboratories, they have reliable whole blood anticoagulation properties that consistently yield high quality plasma, and the nuclease inhibitory properties of the EDTA are ideal for protecting and preserving cfDNA fragments. The cfDNA yields from 84 independent healthy volunteers were quantified and the resulting DNA was analyzed by gel electrophoresis (Fig 3). Nucleosomal fragments were the dominant DNA species observed in all samples, with a representative set of four samples shown in Fig 3A. The average yield of cfDNA was 6.6 ± 3.2 nanograms per mL of plasma (2.2 ng/mL min, 18.0 ng/mL max; Fig 3D and S1 Table). From the perspective of genomic analysis, this translates into ~2000 haploid human genomes per mL of clarified plasma. To determine if the time between whole blood collection and separation of plasma by centrifugation was critical, whole blood collection were maintained at ambient temperatures for variable amounts of time prior to centrifugation (Fig 3B). Moreover, we determined that the modest amounts of gDNA liberated at later time points did not significantly interfere with the performance of the targeted hybrid capture assay used to analyze cfDNA samples (S3 Fig). The yield and integrity of the cfDNAs that were subsequently extracted from clarified plasma were highly similar, suggesting that the time from whole blood collection to separation of the plasma fraction can be delayed for several hours without adverse consequences.

**Fig 4:**
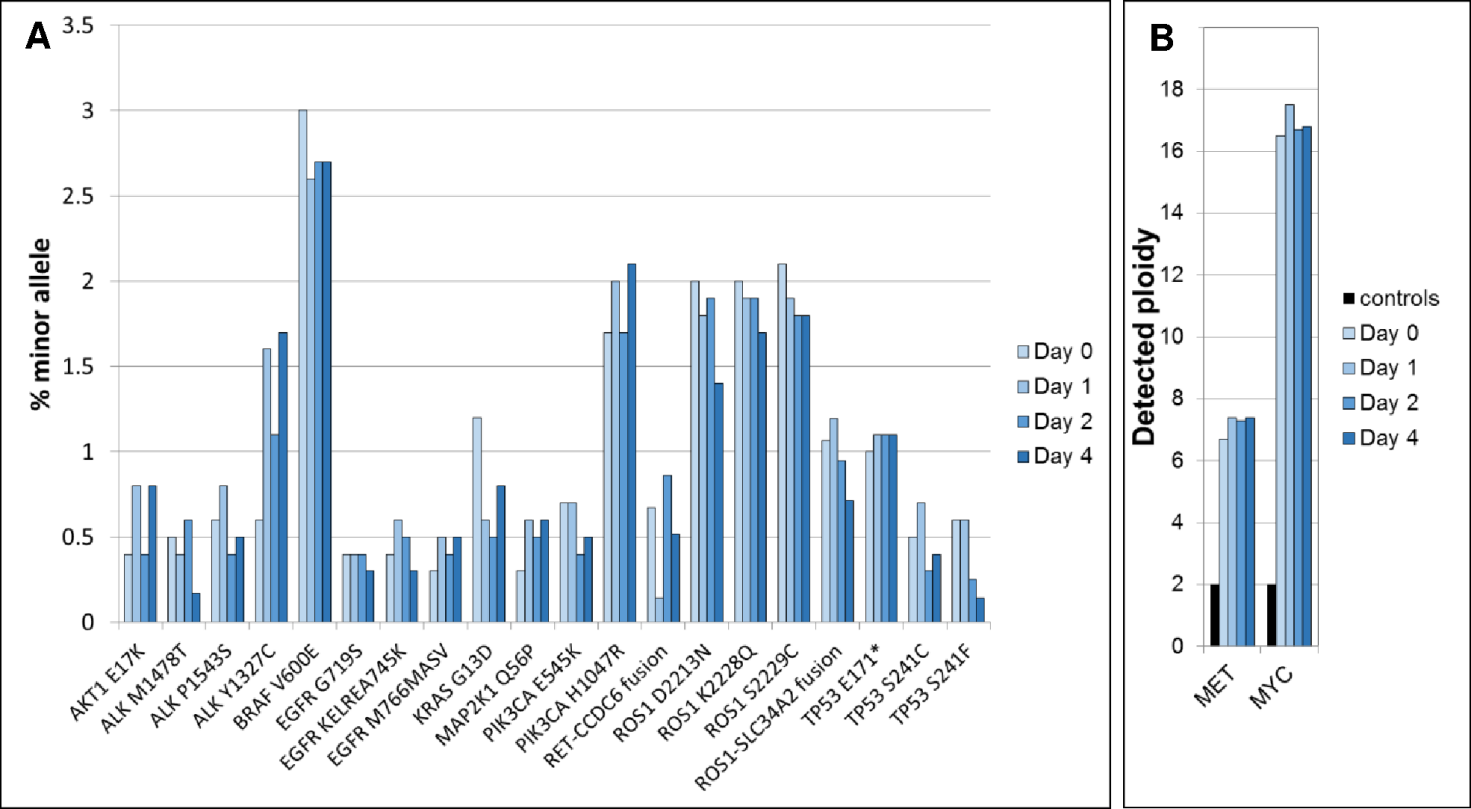
Stability testing of K2 EDTA plasma. (A) Mutation calls in four samples stored for 0, 1, 2 or 4 days at room temperature. The gene panel used detects 20 minor variants in the HD786 structural multiplex cfDNA reference standard, 12 of which are at or below 0.5% minor allele frequency. (B) Detection of copy number amplification in MET and MYC genes as a function of plasma storage times.

In principle, the DNA in clarified plasma that contains EDTA should be stable for several days at ambient temperatures. To test this, clarified plasma was stored on-site for variable amounts of time, then shipped at ambient temperatures using a standard overnight carrier service. The time from plasma preparation to cfDNA extraction varied from 24 hours to 7 days. The DNA yields and fragment integrity extracted from these samples appeared to be highly similar (Fig 3C). Encouraged by the apparent stability of nucleosomal DNA fragments over several days, we were motivated to test the stability of analytical samples that contained cancer-related mutations at minor allele frequencies representative of patient samples (Fig 4). A cfDNA reference standard containing single nucleotide variants, a short insertion and deletion, fusion genes and two copy number amplified genes (Methods) was added to a single, healthy donor plasma sample, and aliquots were stored at room temperature for various times prior to sequencing and analysis. All of the added mutant fragments were detected in every sample, and there was little significant change in the overall yield of sample sequencing depth or in the minor mutant allele frequencies observed in these samples. Importantly, the calls for several different lesion types including single nucleotide variants, insertions/deletions, gene fusions and gene copy amplification were all stable over time. These data raise the possibility K2 EDTA-collected clinical plasma samples intended for cfDNA analysis can be shipped at ambient temperatures.

### High volume collection of plasma

In clinical analysis settings, there are several applications that create the need for the collection of large amounts of cfDNA-containing plasma. First, routine process controls for reproducibility and large analytical validation studies both require large amounts of *bona-fide* plasma from a single source. Second, the sensitivity of residual and relapse disease monitoring in previously treated patients is directly proportional to the quantity of cfDNA analyzed. High volume plasma collection methods can therefore facilitate the highly sensitive monitoring of mutations that may be present at very low allelic frequencies. Third, early detection of cancer-related mutations in healthy individuals has the same constraints as disease monitoring. Large volume plasma collection using plasmapheresis devices is a well-established procedure. To determine if this collection method is suitable for cfDNA clinical procedures, cfDNA was extracted from a total of 40 independent plasmapheresis samples (S2 Table). This procedure yielded an average of 4.4 ng cfDNA per mL of plasma all of which appeared to be intact nucleosomal fragments. These data imply that a typical 800 mL plasma collection will contain the equivalent of approximately one million haploid human genomes. There was little or no evidence of high molecular weight gDNA from lysed blood cells in any of the samples collected using the automated collection instruments. These observations suggest that plasmapheresis is a viable method for collecting large quantities of cfDNA from a single individual.

## Discussion

While much has been written about cell-free DNA, there is conflicting information about which DNA species—high molecular weight DNA or nucleosomal fragments—is of greatest diagnostic relevance. We show examples here where DNA extracted from the plasma of both healthy donors and cancer patients is composed almost exclusively of small fragments. The targeted hybrid capture methods used in this study selectively enrich for fragmented DNA molecules, and examples are shown where solid tumor genetic markers are abundantly represented in the resulting sequencing libraries. For example, in the two patient samples shown in Fig 1C, 32% and 31% of the total reads from the ALK gene region that span the fusion junction were tumor-derived EML4-ALK fusion sequences. Others have also reported that the circulating, tumor-derived DNA are generally short and highly fragmented molecules [9,16]. These observations have prompted us to focus on collection, shipping and analysis methods that preserve the nucleosomal DNA fraction in plasma samples.

Where does high molecular weight genomic DNA come from and how does it affect genomic analysis? One source is inadvertent inclusion of nucleated buffy coat cells during plasma purification. We describe a double-spin protocol (Materials and Methods) that largely eliminates high molecular weight genomic DNA from cfDNA preparations. We also show that hybrid capture protocols that include a library construction step essentially eliminate high molecular weight DNA from subsequent analysis (S3 Fig). The same cannot be said of technologies that rely on PCR amplification of target genomic loci. In these cases, high molecular weight DNA from blood cells will contribute to the detected fraction of germ-line sequence reads and diminish estimates of somatic tumor mutation minor allele frequencies. This artifact could be particularly problematic in longitudinal monitoring applications where estimates of disease burden can influence patient treatment decisions.

Recent papers have demonstrated the influence of nucleosome phasing on the distribution of cfDNA fragmentation patterns [10,17]. The nucleotide bias we observed on the clone ends of cfDNA from germline and tumor fragments has been reported previously [17], and is presumably a signature of the endonuclease activities present in the human body. In addition to favored motifs at cfDNA cleavage sites (e.g. CC dinucleotides) we also observe cleavage “hotspots” adjacent most of our DNA capture probes, and these hotspots appear to be a reflection of phased nucleosomes [10,17]. From a clinical laboratory perspective where analytical validation studies are required, it is not routinely plausible to obtain genuine cfDNA from well-characterized standards. Currently, commutability studies of the type shown in Fig 4 where sonicated standards are spiked into plasma-derived cfDNA are necessary. It will be a major breakthrough in the standardization of cfDNA genomic assay performance if the DNA released into the media of cultured cells [18] bears any resemblance to the cfDNA extracted from plasma.

There is an extensive literature on methods to collect and ship samples that contain cfDNA e.g. [19,20]. Cell-Free DNA BCT tubes manufactured by Streck (La Vista, NE) are frequently used because they enable whole blood to be shipped from the site of collection to the site of specimen processing. Our laboratory has had good results with specimens collected in these tubes. However, conventional K2 EDTA collection tubes are a more ubiquitous and less expensive option. Here we provide preliminary data that the cfDNA present in K2 EDTA clarified plasma is stable at ambient temperatures for several days. While more extensive stability studies are needed, the use of reasonably priced tubes that are approved for diagnostic procedures coupled with ambient temperature shipping may substantially ease the logistical burden associated with sample collection and transport. Finally, we demonstrate the high quality cfDNA is present in the plasma collected by high volume plasmapheresis collection devices. The ability to collect abundant quantities of cfDNA from a single individual will facilitate clinical operations, minimal disease monitoring, and early detection of cancer.

## Supporting information

S1 Fig Summary of the Resolution Bioscience hybrid capture technology. (A) Genomic library construction specifically enriches for nucleosomal fragment clones. Adaptors that enable amplification, unique molecule identification and sample multiplexing are used to create genomic libraries. (B) Denatured library is hybridized with tailed capture probes. The probe sequence is then extended to copy the genomic insert and adaptor sequence. (C) The sequence of genomic clones are determined using asymmetric, paired-end sequencing.
(PPTX)

S2 Fig The base composition of the first 25 bases from 2,241 unique EML4-ALK fusion clones. The EML4 gene is relatively A/T rich and certain cfDNA cleavage sites are highly favored. This results in a “noisy” plot of base composition.
(PPTX)

S3 Fig Contaminating high molecular weight gDNA does not significantly interfere with detection of low frequency markers in a non-reference standard. **(A)** Twenty nanograms of a non-reference standard containing 17 SNVs and 5 indels at 1% MAF was made into a genomic library (control “Reference sample”). An identical amount was combined with 100 ng of high molecular weight gDNA and also made into a library (Spike-in). Targeted hybrid capture, sequencing and standard bioinformatics pipeline analysis were used to measure the detection rates and minor allele frequencies of these markers. The spike-in gDNA had a unique SNV that allowed direct determination of its fraction in the overall library.
(PPTX)

S1 Table Yield of cfDNA from 84 K2 EDTA tubes collected from healthy volunteers. (XLSX)

S2 Table Yield of cfDNA from 40 high-volume plasmapheresis samples. (XLSX)
