# Supplementary material for: Collection of cell-free DNA for genomic analysis of solid tumors in a clinical laboratory setting
Source: PLoS One. 2017 Apr 27;12(4):e0176241. doi: 10.1371/journal.pone.0176241 (PMC5407747; doi:10.1371/journal.pone.0176241)
Supplement: S1 Fig — (A) Genomic library construction specifically enriches for nucleosomal fragment clones. Adaptors that enable amplification, unique molecule identification and sample multiplexing are used to create genomic libraries. (B) Denatured library is hybridized with tailed capture probes. The probe sequence is then extended to copy the genomic insert and adaptor sequence. (C) The sequence of genomic clones are determined using asymmetric, paired-end sequencing. (PPTX) [file pone.0176241.s001.pptx]

## Slide 1
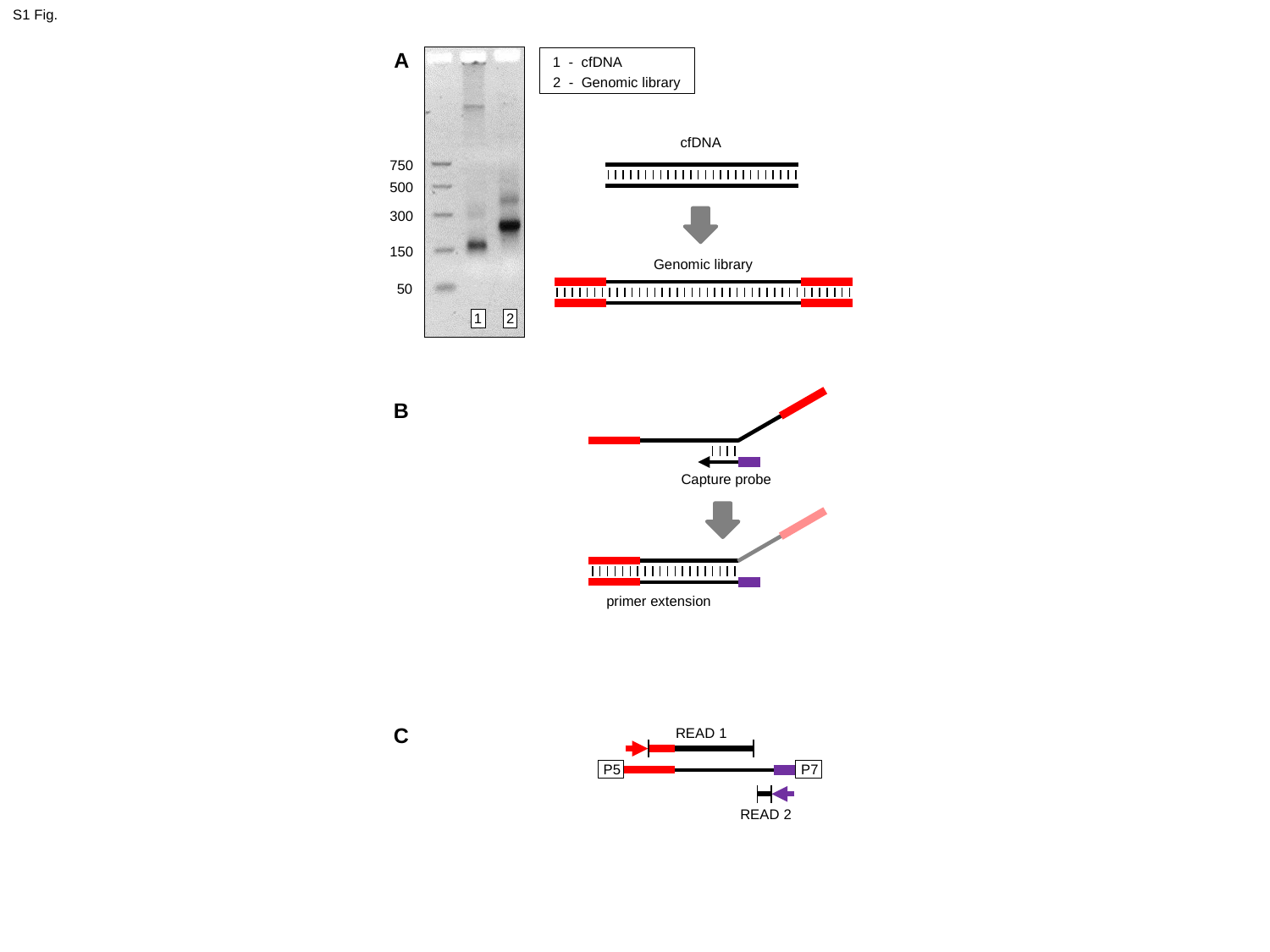

S1 Fig.
A
1 - cfDNA
2 - Genomic library
cfDNA
750
500
300
150
Genomic library
50
1
2
B
Capture probe
primer extension
C
READ 1
P5
P7
READ 2
