# Supplementary figures and images for: Collection of cell-free DNA for genomic analysis of solid tumors in a clinical laboratory setting
Source: PLoS One. 2017 Apr 27;12(4):e0176241. doi: 10.1371/journal.pone.0176241 (PMC5407747; doi:10.1371/journal.pone.0176241)

## Slide 1
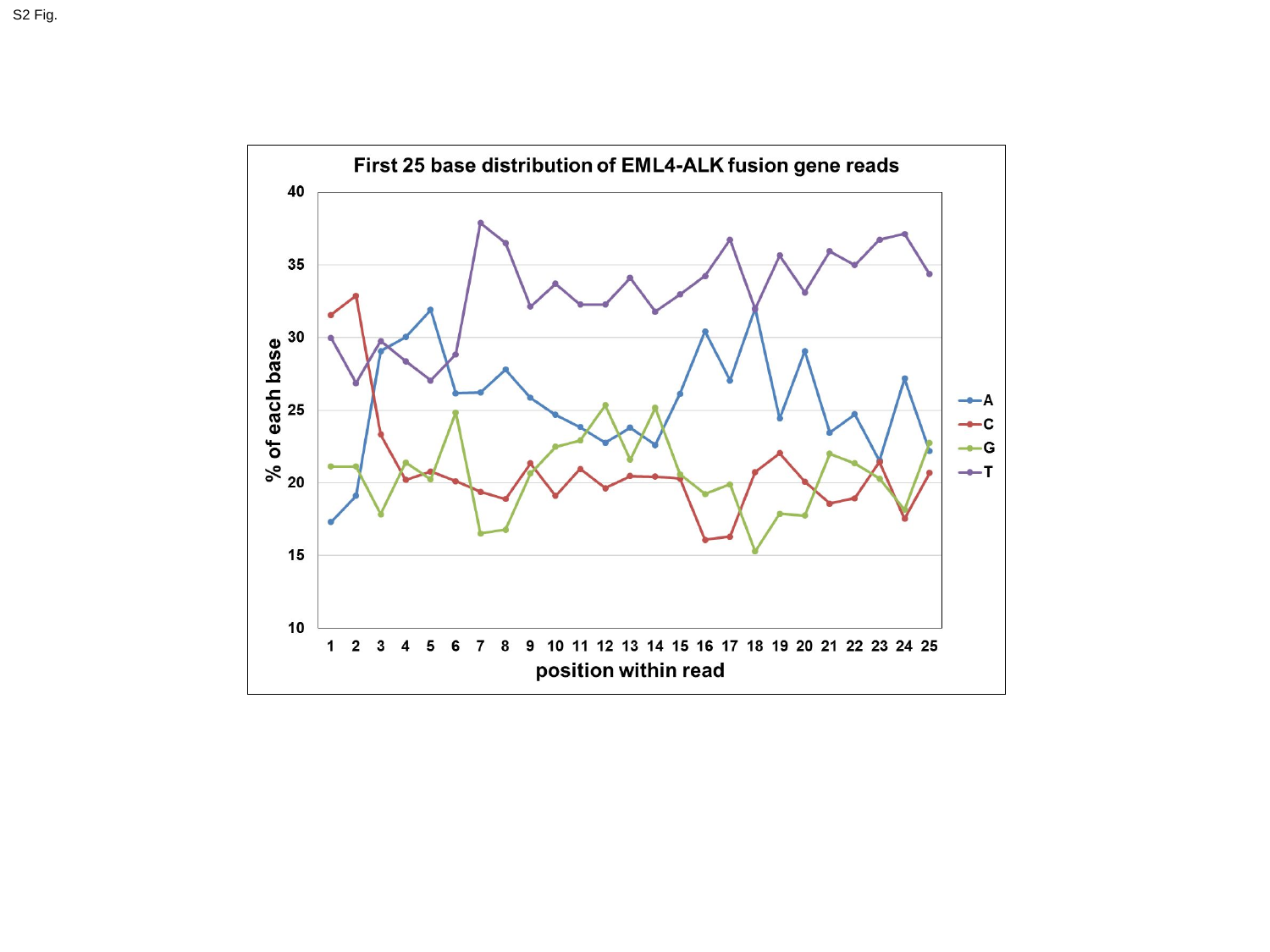

S2 Fig.

Supplement: S2 Fig — The EML4 gene is relatively A/T rich and certain cfDNA cleavage sites are highly favored. This results in a “noisy” plot of base composition. (PPTX) [file pone.0176241.s002.pptx]

## Slide 1
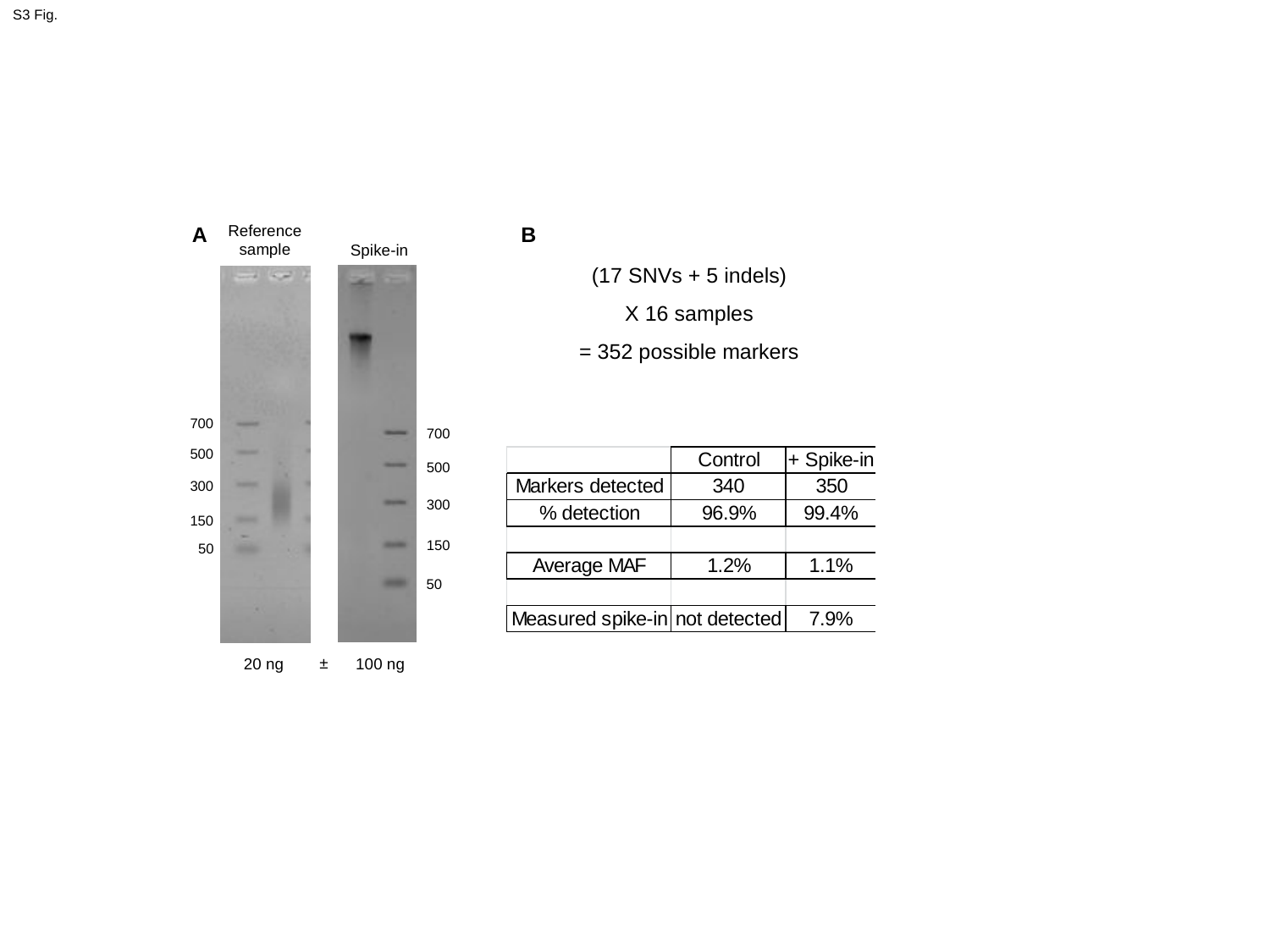

S3 Fig.
Reference
sample
A
B
Spike-in
(17 SNVs + 5 indels)
X 16 samples
= 352 possible markers
700
700
500
500
300
300
150
150
50
50
±
20 ng
100 ng

Supplement: S3 Fig — (A) Twenty nanograms of a non-reference standard containing 17 SNVs and 5 indels at 1% MAF was made into a genomic library (control “Reference sample”). An identical amount was combined with 100 ng of high molecular weight gDNA and also made into a library (Spike-in). Targeted hybrid capture, sequencing and standard bioinformatics pipeline analysis were used to measure the detection rates and minor allele frequencies of these markers. The spike-in gDNA had a unique SNV that allowed direct determination of its fraction in the overall library. (PPTX) [file pone.0176241.s003.pptx]
